# Genetic and physical localization of a major susceptibility gene to *Pyrenophora teres* f. *maculata* in barley

**DOI:** 10.1007/s00122-023-04367-1

**Published:** 2023-04-27

**Authors:** Abdullah F. Alhashel, Jason D. Fiedler, Raja Sekhar Nandety, Ryan M. Skiba, Robert S. Bruggeman, Thomas Baldwin, Timothy L. Friesen, Shengming Yang

**Affiliations:** 1 Department of Plant Pathology, North Dakota State University, Fargo, ND 58102 USA; 2 Department of Plant Protection, College of Food and Agriculture Sciences, King Saud University, Riyadh, 11451 Saudi Arabia; 3 Department of Plant Sciences, North Dakota State University, Fargo, ND 58102 USA; 4 Cereals Crops Research Unit, Edward T. Schafer Agricultural Research Center, USDA-ARS, Fargo, ND 58102 USA; 5 Department of Crop and Soil Sciences, Washington State University, Pullman, WA 99164 USA

## Abstract

**Key message:**

Genetic characterization of a major spot form net blotch susceptibility locus to using linkage mapping to identify a candidate gene and user-friendly markers in barley.

**Abstract:**

Spot form net blotch (SFNB), caused by the necrotrophic fungal pathogen *Pyrenophora teres* f. *maculata* (*Ptm*), is an economically important foliar diseases in barley. Although various resistance loci have been identified, breeding for SFNB-resistant varieties has been hampered due to the complex virulence profile of *Ptm* populations. One resistance locus in the host may be effective against one specific isolate, but it may confer susceptibility to other isolates. A major susceptibility QTL on chromosome 7H, named *Sptm1*, was consistently identified in many studies. In the present study, we conduct fine mapping to localize *Sptm1* with high resolution. A segregating population was developed from selected F_2_ progenies of the cross Tradition (S) × PI 67381 (R), in which the disease phenotype was determined by the *Sptm1* locus alone. Disease phenotypes of critical recombinants were confirmed in the following two consecutive generations. Genetic mapping anchored the *Sptm1* gene to an ⁓400 kb region on chromosome 7H. Gene prediction and annotation identified six protein-coding genes in the delimited *Sptm1* region, and the gene encoding a putative cold-responsive protein kinase was selected as a strong candidate. Therefore, providing fine localization and candidate of *Sptm1* for functional validation, our study will facilitate the understanding of susceptibility mechanism underlying the barley-*Ptm* interaction and offers a potential target for gene editing to develop valuable materials with broad-spectrum resistance to SFNB.

**Supplementary Information:**

The online version contains supplementary material available at 10.1007/s00122-023-04367-1.

## Introduction

Barley (*Hordeum vulgare* L.) is an important cereal crop in the grass family Poaceae. It is the fourth largest cereal crop in both yield and cultivation area globally, after wheat, rice, and corn (Poehlman 1987). Barley is primarily grown for animal feed and malt which is important in the brewing and distilling industries. In some developing countries, it is also a staple food for human consumption. However, barley production is seriously threatened by various diseases. One of the important foliar diseases in barley is net blotch (NB) caused by the fungal pathogen *Pyrenophora teres*, resulting in up to 10–40% yield losses when susceptible varieties are grown (Mouchacca 1999).

Net blotch occurs in two forms, spot form (SFNB) and net form (NFNB) caused by *P. teres* f. *maculata* (*Ptm*) and *P. teres* f. *teres* (*Ptt*), respectively. Although hybrids can be obtained in the laboratory, *Ptm* and *Ptt* are genetically distinct, and they induce different symptoms on susceptible hosts (Liu et al. 2011). Diagnostic markers have been developed to differentiate the two forms of *P. teres* (Leisova et al. 2006). Moreover, *Ptt* infects as a necrotroph and grows mostly in the apoplast. In contrast, *Ptm* initially forms intracellular vesicles near the penetration site before switching to intercellular growth resulting in necrosis (Lightfoot and Able 2010). The early laten phase indicated that *Ptm* may secrete additional effectors to suppress host defense responses at the initial infection stage (Whisson et al. 2007). Both forms of *P. teres* secreted phytotoxins or necrotrophic effector responsible for the necrosis and chlorosis, while *Ptt* produces significantly more toxins in the culture medium (Lightfoot and Able 2010; Sarpeleh et al. 2007, 2008). Many of these host specific toxins or necrotrophic effectors are proteinaceous (Sarpeleh et al. 2007). Hijacking the host defense in an inverse gene-for-gene manner, necrotrophic effectors manipulate host susceptibility proteins or targets to induces programmed cell death (PCD) for necrotrophs to acquire nutrients from destroyed cells (Friesen and Faris 2021).

SFNB has been increasingly damaging in barley growing regions (Liu et al. 2011). Harnessing genetic resistance is an effective and sustainable means for disease control. Nevertheless, due to the sexual recombination in *Ptm* populations, the rapid evolution of effectors diversifies the virulence profiles. As a result, host reactions to *Ptm* are complex and controlled by various quantitative trait loci (QTL). Additionally, a QTL effective against certain isolates may be susceptible to others. Genome-wide association and linkage mapping studies have identified only a few major QTL which have been consistently detected using various *Ptm* isolates, including *Rpt4* (on chromosome 7H), *Rpt5* (6H), *Rpt6* (5H), *Rpt7* (4H), and *Rpt8* (4H) (Alhashel et al. 2021; Daba et al. 2019; Franckowiak and Platz 2013; Friesen et al. 2006; Grewal et al. 2008; Manninen et al. 2006; Raman et al. 2003; Richards et al. 2016; Tamang et al. 2019; Vatter et al. 2017; Yun et al. 2005). These QTL provide valuable resources for breeding broad-spectrum resistance to SFNB. However, the identity and functional mechanisms of the genes underlying these QTL have been elusive.

The *Rpt4* locus is strikingly important among the major QTL. This locus confers broad-spectrum resistance/susceptibility to *Ptm* including some isolates with unique virulence profiles, and it is effective against multiple *Ptt* isolates as well (Alhashel et al. 2021; Daba et al. 2019; Duellman 2015; Grewal et al. 2008; Wonneberger et al. 2017). Furthermore, although *Rpt4* was identified as a dominant seedling resistance, it also contributed to adult plant resistance (APR) (Williams et al. 1999). The broad specificity of *Rpt4* was confirmed by Tamang et al. (2019) using six geographically distinct isolates, but segregation ratios in the biparental population suggested that *Rpt4* conditioned dominant susceptibility to SFNB (Tamang et al. 2019). Recent research in barley-*Ptm* interactions identified two major virulence loci located on *Ptm* Chr1 and Chr2, respectively, with the *Ptm* virulence on Chr2 targeting a dominant susceptibility gene at the *Rph4* locus on barley 7H (Skiba et al. 2022). An inverse gene-for-gene association was demonstrated by the host and pathogen genetics in the barley-*Ptm* pathosystem (Skiba et al. 2022).

In the present study, we conducted genetic and physical mapping to identify the gene underlying the *Rph4* locus. To avoid misperception, the gene is designated *Susceptibility to Ptm 1* (*Sptm1*) hereafter. Genetic mapping delimited the *Sptm1* gene within a ⁓400 kb region on 7H. A total of six protein-coding genes were identified in the *Sptm1* region. Of those, one gene encoding a putative protein kinase was selected as a promising candidate for functional validation. Therefore, our research lays a foundation to isolate this agronomically and genetically important *Sptm1* gene, which will facilitate our understanding of the molecular mechanisms regulating the barley-*Ptm* interactions and provide a target for gene manipulation to develop SFNB-resistant resources.

## Materials and methods

### *Ptm* isolate and plant materials

*Ptm* isolate Cel-A17 (CA17) collected in Montana State was used to map *Sptm1* in this study. Using a recombinant inbred line (RIL) population derived from the cross between Tradition (six-rowed, susceptible) and PI 67,381 (two-rowed, resistant). Tamang et al. (2019) identified a total of three QTL against CA17 including the susceptibility gene *Sptm1* on 7H. The other two QTL were located on 2H (*QRptm-2H-1–31*) and 3H (*QRptm-3H-81–88*) (Tamang et al. 2019). Using SNP markers flanking these three loci, we identified six plants from 200 Tradition × PI 67,381 F_2_ lines that were heterozygous for *Sptm1* and homozygous recessive for *QRptm-2H-1–31* and *QRptm-3H-81–88*. Segregating populations used to map *Sptm1* were developed by selfing the selected six F_2_ plants. A total of 702 F_2:3_ plants were used for genetic mapping. Critical recombinants were assessed in the next generation with at least 40 F_3:4_ plants, and the derived homozygous F_3:4_ recombinant representing immortal critical recombinant (ICR) were used to increase seeds for further phenotyping. At least 30 ICRs (F_4:5_) were used to confirm the phenotype for each original F_2:3_ recombinant.

### Inoculum preparation and phenotyping

The CA17 inoculum preparation, inoculation, and phenotyping were conducted as described by Neupane et al. (2015). Briefly, spores were collected with sterilized distilled water from V8-PDA culture plates (150 ml V8 juice, 10 g Difco PDA, 3 g CaCO_3_, 10 g agar, and 850 ml H_2_O). The spore concentration was adjusted to 2000 spores/ml with two drops of Tween-20 per 100 ml added. Barley segregants together with Tradition and PI 67,381 were individually grown in super-cell cones placed in RL98 trays. Inoculation was performed when the second leaf was fully expanded (∼2 weeks) using an air sprayer at 15 to 20 psi. The inoculated plants were kept in a mist chamber at 100% relative humidity for 24 h under continuous light, and then moved to a growth chamber under a 12 h/12 h—light/dark cycle at 21 °C. Disease reactions were assessed 7 days post inoculation (DPI) using a 1–5 rating scale with 1 being highly resistant and 5 being highly susceptible (Neupane et al. 2015).

Barley genotypes used for pangenome sequencing were also tested for disease responses with at least eight plants for each line (Jayakodi et al. 2020). Two plants in each cone were scored collectively as a single replicate, and at least four independent replicates for each genotype were conducted. The average value of all replicates was used as the phenotypic score.

### Genotyping, marker development, and linkage mapping

The CTAB protocol was used to extract DNA (Murray and Thompson 1980). Around 100 mg of leaf tissue were collected from plants at the three-leaf stage. DNA concentration was quantified using a NanoDrop spectrophotometer (NanoDrop 8000, Thermo Fisher Scientific) according to the manufacturer’s instructions. The parental lines Tradition and PI 67,381 were genotyped using the barley 50 k iSelect SNP Array to identify markers (Bayer et al. 2017). GenomeStudio V2.0 (Illumina) was used for genotype calling with the de novo calling algorithm. The called SNPs, together with those flanking *Sptm1*, *QRPtm-2H-1–31* (2H), and *QRPtm-3H-81–88* (3H) reported by Tamang et al. (2019), were converted to semi-thermal asymmetric reverse PCR (STARP) markers (Table S1) (Long et al. 2017). PCR protocol and conditions were followed as previously described (Long et al. 2017). Amplicons were assayed on a 6% polyacrylamide gel stained with GelRed™ (MilliporeSigma), which was imaged using a Typhoon™ FLA 9500 variable mode laser scanner (GE Healthcare Life Sciences, Marlborough, MA). Simple sequence repeats (SSRs) markers were also developed using the barley cv. Morex v3 reference assembly (Mascher et al. 2017).

More SNPs were obtained to saturate the *Sptm1* genetic region by genome re-sequencing of Tradition and PI 67,381. Paired-end sequencing of the genomic libraries was performed on an Illumina Novaseq 6000 system with 150 bp paired ends. All the Illumina paired end reads were cleaned with bbduk using the following parameters, ktrim = r; *K* = 23; mink = 11 and hdist = 1 (Bushnell 2021). The cleaned reads are aligned to the Morex v3 reference genome, and alignment files were then sorted and indexed using SAMtools (Danecek et al. 2021; Langmead et al. 2009). Filtered with a minimum mapping quality score of 30 and a minimum reads depth coverage of 4, raw single nucleotide variants and indels were called using SAMtools and bcftools v1.14, (Li 2011). SNP and indel variants were annotated with BEDtools (Quinlan and Hall 2010).

Genetic map was constructed using JoinMap 3.0 (Stam 1993). All markers used for genetic mapping of *Sptm1* are listed in Table S2.

### Physical mapping and sequence analysis of candidate genes in the *Sptm1* region

The programs FGENESH and Pfam 32.0 were used to perform gene prediction and annotation. The predicted gene structure was verified using BaRTv1.0, a high-quality, non-redundant barley reference transcripts database (Barley Reference Transcripts–BaRTv1.0) (Rapazote-Flores et al. 2019). We extracted the fasta sequences of the annotated genes from the Morex v3 genome assembly corresponding to the *Sptm1* region (Chr7H:592,631,221–593,037,317). With extra 1000 bp flanking coding region for each of the annotated gene. Genome re-sequencing reads of parental lines were mapped to the Morex v3 genome, and the aligned bam files were subset to the *Sptm1* region using bcftools to generate variant call format files (VCFs). The generated variant files were visualized along with the bam files using Integrative Genomics Viewer (IGV) to identify sequence polymorphisms between alleles.

## Results

### Population development and phenotype evaluation

**Fig. 1:**
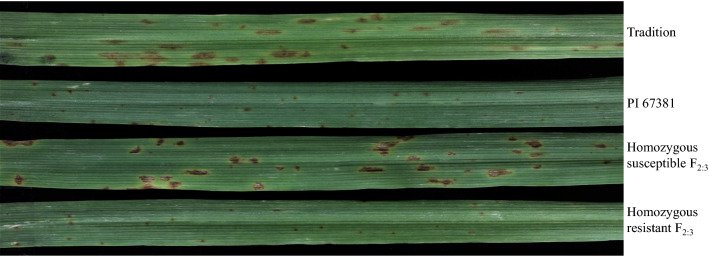
Phenotypic responses of parental lines Tradition and PI 67,381 and homozygous F_2:3_ plants to *Ptm* isolate CA17. Typical SFNB symptom with large necrotic lesions was shown in Tradition homozygous susceptible F_2:3_ plant, while PI 67,381 and homozygous resistant F_2:3_ plant were incompatible with the pathogen

Tamang et al. (2019) identified 3 QTL associated with susceptibility to *Ptm* isolate CA17 using RILs of Tradition (S) × PI 67,381 (R), including *Sptm1*, *QRptm-2H-1–31* (2H) and *QRptm-3H-81–88* (3H). Using the SNP markers flanking these three QTL (Table S1), we excluded the genetic effect of QTL on 2H and 3H, and the disease phenotype in the resulting F_2:3_ population was controlled by *Sptm1* only. Besides the resistant and susceptible F_2:3_ extremes, we identified a large number of plants showing intermediate disease types. F_2:3_ plants carrying homozygous PI 67,381 allele (*n* = 15), indicated by *Sptm1*-flanking SNP markers, exhibited an average disease reaction of 1.38 which was not significantly different from the score of PI 67,381 (1.5) (Table 1). F_2:3_ plants carrying homozygous Tradition allele (*n* = 15) showed typical SFNB symptoms 7 DPI with large necrotic lesions surrounded by a chlorotic halo on infected leaves, although their average disease type (3.3) was lower that of Tradition (4.5) (Fig. 1, Table 1). Therefore, based on the disease types of homozygous segregants, plants were considered to be resistant, intermediate, and susceptible if they displayed a reaction type ≤ 1.5, ≥ 2 but < 3, and ≥ 3, respectively. Phenotyping of an initial 178 F_2:3_ plants identified 35 resistant, 97 intermediate, and 46 susceptible. The segregation ratio fits 1:2:1 (*χ*^2^ = 2.80, d*f* = 2, *P* = 0.25), suggesting that the disease reaction is controlled by a single gene with dosage effect.

**Table 1:** Phenotype analysis with Tradition, PI67381, and homozygous F2:3 plants

Genotype	Average reaction type
PI 67,381	1.5^a^ ± 0
Tradition	4.53^c^ ± 0.41
F_2:3_ with homozygous PI 67,381 allele of *Sptm1*	1.38^a^ ± 0.44
F_2:3_ with homozygous Tradition allele of *Sptm1*	3.30^b^ ± 0.51

Disease severity for each genotype was indicated by the average reaction type and standard deviation. The average values with different letters were significantly different at the 0.05 level of probability using Tukey test

### Genetic and physical mapping

**Fig. 2:**
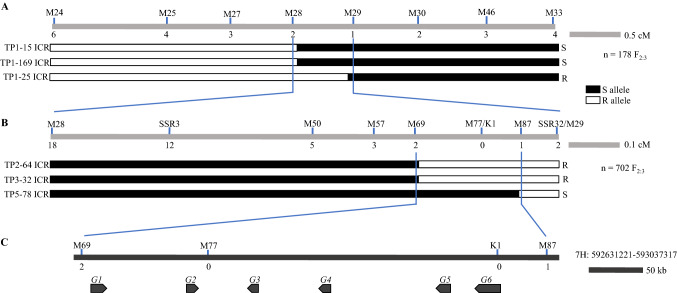
Fine mapping of *Sptm1*. Genetic mapping was conducted sequentially with 178 (**A**) and 524 (**B**) F_2:3_ individuals representing 356 and 1048 gametes, respectively. Phenotypes of critical recombinants were first confirmed with F_3:4_ plants from which ICRs were selected. At least 30 ICRs for each recombinant were also tested to verify the disease response. The ICRs used to delimit the *Sptm1* gene are shown by a combination of differential boxes. Black box represents homozygous susceptible genotype, and empty for homozygous resistant. Numbers below the linkage group indicate the number of recombination breakpoints separating the marker from *Sptm1*. A total of six protein-coding genes were identified in the *Sptm1* region spanning ~ 400 kb (**C**). The maps are drawn to scale. M, marker; ICR, immortal critical recombinant; R, resistant; S, susceptible; G, gene

SNP markers on 7H were identified through genotyping of parental lines using the barley 50 k SNP array (Table S2). Genetic mapping of *Sptm1* was first conducted with the 178 F_2:3_ plants. Using 81 resistant and susceptible F_2:3_ extremes, we initially delimited the *Sptm1* gene to a 6 Mb region flanked by SNP markers M24 and M33 (Fig. 2A). Plants including intermediate individuals genotyped as recombinants by M24 and M33 were saved for seed increase. All recombinants went through phenotype confirmation using both F_3:4_ plants and the derived homozygous ICRs. Relying on the ICRs of critical recombinants TP1-15 (the 15th plant in batch 1 tested), TP1-169, and TP1-25, the *Sptm1* region was narrowed down to 1.2 Mb flanked by M28 and M29 (Fig. 2A). To increase the mapping resolution, we enlarged the population to 702 F_2:3_ individuals (Fig. 2B). More SNPs were called using short-read sequencing of Tradition and PI 67,381 to saturate the *Sptm1* region (Table S2). A total of 20 recombinants were identified between M28 and M29. Taking advantage of the same phenotyping strategy involving F_3:4_ plants and ICRs, the *Sptm1* gene was finally delimited to an ⁓400 kb region (Chr7H: 592,631,221–593,037,317) flanked by M69 and M87 based on three critical recombinants TP2-64, TP3-32, and TP5-78 (Fig. 2B).

Gene annotation and prediction identified a total of six putative protein-coding genes according to the Morex v3 genome assembly (Fig. 2C, Table 2). Of those, four genes (*G1*–*G4*) encode either hypothetical or uncharacterized proteins (Table 2). The coding product of *G5* (*HORVU.MOREX.r3.7HG0735550*) is homologous to human protein Werner Syndrome Exonuclease (WEX) with an exonuclease domain. *G6* (*HORVU.MOREX.r3.7HG0735560*) encodes a protein with homology to Cold-responsive Protein Kinase 1 (CRPK1, At1G16670) in *Arabidopsis thaliana* (Table 2) (Liu et al. 2017). Because protein kinases play crucial roles in various signal transduction cascades, particularly in plant–microbe interactions, we focused on *G6* for further analysis.

**Table 2:** Predicted genes in the *Sptm1* region. *G*, gene

Gene	Gene ID	Homology
*G1*	*HORVU.MOREX.r3.7HG0735510*	Hypothetic protein with unknown function
*G2*	*HORVU.MOREX.r3.7HG0735520*	No homology
*G3*	*HORVU.MOREX.r3.7HG0735530*	No homology
*G4*	*HORVU.MOREX.r3.7HG0735540*	Hypothetic protein with unknown function
*G5*	*HORVU.MOREX.r3.7HG0735550*	Werner syndrome-like exonuclease
*G6*	*HORVU.MOREX.r3.7HG0735560*	Cold-responsive protein kinase 1

### Allelic polymorphisms between resistant and susceptible *G6* alleles

**Fig. 3:**
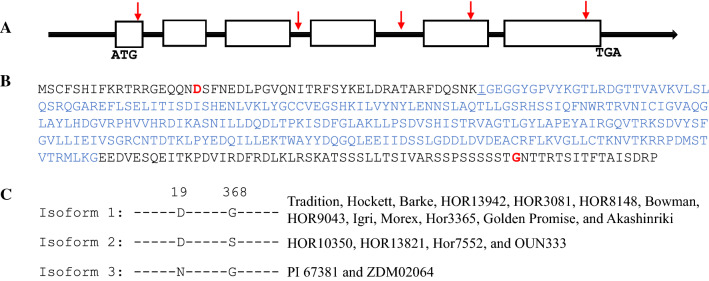
Sequence analysis of *G6*. The coding region of *G6* contains 6 exons and 5 introns (**A**), encoding a protein of 384 aa (B). Exons are shown as empty boxes, and black line for introns. The SNPs identified between Tradition and PI 67,381 alleles are indicated by red arrows. The catalytic domain of protein kinase is highlighted in blue. The aa substitutions identified among allele products are highlighted in red (**B**). Three G6 isoforms were identified using pangenome references and Bowman, and the D19N substitution is associated with disease type (**C**)

The coding region of *G6* contains 6 exons and 5 introns, encoding a protein of 384 amino acids (aa) with a molecular weight of 42.8 kDa, composed of the catalytic domain of serine/threonine-specific and tyrosine-specific protein kinases (Fig. 3A). Allelic sequences of *G6* were obtained by mapping WGS reads to the Morex v3 genome reference. A total of five SNPs, three in exons and two in introns, were identified between Tradition and PI 67,381 alleles, resulting in only one aa substitution D19N at the N-terminus (Fig. 3A–B). All SNPs were confirmed by Sanger sequencing as well.

The pangenome derived from 20 barley accessions representing the global diversity provides an important tool to reveal the hidden allelic variations (Jayakodi et al. 2020). To investigate if the protein haplotype is associated with disease phenotype, we assessed 17 available pangenome references and a transformable variety Bowman. The result showed that 17 accessions including Morex were susceptible or intermediately susceptible, but ZDM02064 (Chiba) was the only line resistant to CA17 (Fig. 3C, Table S3). Two protein isoforms were identified among the susceptible lines, distinguished by an aa substitution at the distal C-terminus, G368S. Most susceptible lines (13 lines) carry the same protein isoform as Tradition (Fig. 3C). Notably, although the aa substitution D19N is outside of the conserved functional domain (Fig. 3B), the resistant line ZDM02064 shares the same protein haplotype as the resistant parental line PI 67,831 (Fig. 3C), which therefore strengthens the candidacy for this protein kinase gene.

## Discussion

SFNB has been increasingly damaging for barley production. Genetic resistance has been identified in barley and its wild relatives, and the introgression of resistance from various sources into barley may facilitate achievement of more effective and durable resistance. However, the complex and quantitative nature of host responses has posed a major challenge to deploying effective and durable resistances (Wang et al. 2015), which also limits the understanding of molecular mechanisms controlling barley-*Ptm* interactions. Conferring broad spectrum recognition to both *Ptm* and *Ptt*, the *Sptm1* locus provides a valuable resource for breeders and geneticists in barley improvement and genetic studies (Alhashel et al. 2021; Skiba et al. 2022; Tamang et al. 2019; Williams et al. 1999, 2003). In the present research, we conducted genetic and physical mapping toward cloning of the molecular determinant of *Sptm1*. Using ICRs derived from selected F_2_ lines of Tradition × PI 67,381, we precisely anchored the *Sptm1* gene to an ⁓400 kb region on 7H, and *G6* (*HORVU.MOREX.r3.7HG0735560*) homologous to *AtCRPK1* was identified as a promising candidate. In addition, the SNP identified between resistant and susceptible alleles can be used as a diagnostic marker to assist breeding selection.

In a joint genetic analysis of *Ptm* virulence and host susceptibility, Skiba et al. (2022) reported that the *Sptm1* allele of Hockett on 7H interacted with the virulence locus on *Ptm* Chr2 in a inverse gene-for-gene pattern. Of the six putative genes in the *Sptm1* region, *G6* is the only one whose coding product are known to be involved in protein–protein interactions and signal transduction. Although a transmembrane domain for signal sensing is missing in G6, several kinases containing only the catalytic domain have been identified to function in responses to plant pathogens, such as the tomato bacterial speck resistance gene *Pto* (Martin et al. 1993), wheat powdery mildew resistance gene *Pm21* (Cao et al. 2011), wheat stripe rust resistance gene *Yr15* (Klymiuk et al. 2018), wheat stem rust resistance gene *Sr60* (Chen et al. 2020), barley stem rust resistance gene *Rpg1* (Brueggeman et al. 2002), and wheat septoria nodorum blotch susceptibility gene *Snn3* (Zhang et al. 2021). It is noteworthy that Pto, lacking a transmembrane domain, interacts directly with the corresponding avirulence factor avrPto (Frederick et al. 1998). Therefore, under these scenarios, *G6* encoding a protein kinase was designated a strong candidate for *Sptm1*.

An intriguing question is how a protein homologous to AtCRPK1 is involved in plant–microbe interactions. Loss-of-function mutation in *AtCRPK1* results in increased cold tolerance in *Arabidopsis thaliana* (Liu et al. 2017). Located on the plasma membrane, AtCRPK1 phosphorylated 14–3–3 proteins, and the phosphorylated 14–3–3 proteins translocate from cytosol to the nucleus where they destabilize the key cold-responsive C-repeat-binding factor (CBF) proteins. In line with this, overexpression of 14–3–3 enhanced freezing tolerance, while mutations in 14–3–3 improved freezing tolerance. The prominent role of CBF proteins in cold acclimation has been extensively characterized, but there is no precedent for CBFs being involved in plant responses to biotic stress (Shi et al. 2018; Yamaguchi-Shinozaki and Shinozaki 2006). In contrast, 14–3–3 proteins, acting as sensors for the phosphorylation status at specific sites, play significant roles in plant–pathogen interaction as the targets of pathogen effectors or interacting with defense-related proteins (Oh and Martin 2011; Teper et al. 2014; Yang et al. 2009). Therefore, 14–3–3 proteins may be the potential linker between the putative CRPK encoded by *G6* and the susceptibility to *Ptm* pathogen.

The aa substitution D19N associated with disease phenotype is outside the conserved functional domain (CFD) in G6 (Fig. 3), but it is not uncommon that aa substitutions beyond the CFD disrupt protein function (Li et al. 2021, 2016). The specific localization of AtCRPK1 on the plasma membrane indicated the presence of a signal peptide in the protein, although an obvious signal peptide is missing (Liu et al. 2017). The N-terminal sequence of G6 harboring the D19N substitution is homologous to that in AtCRPK1. There may be an uncharacterized signal peptide at the N-terminus, and the aa substitution disturbs protein localization.

In summary, as one of the few genes conferring broad recognition specificity to *Ptm*, *Sptm1* is valuable for variety improvement and fundamental research in barley. The high-resolution mapping in this study provides user-friendly markers and a candidate gene for *Sptm1*. Cloning of *Sptm1* will unravel the genetic mechanism underlying barley susceptibility to this important fungal pathogen, and it will provide a target for gene editing to develop resistant materials. Moreover, it is interesting to investigate if *Sptm1* is also involved in cold tolerance. Nevertheless, the candidate of *Sptm1* will be functionally validated with genetic transformation in barley.

## Supplementary Information

Below is the link to the electronic supplementary material.

Supplementary file1 (PPTX 43 KB)

## Data Availability

The re-sequencing data in this manuscript have been deposited in NCBI Sequence Read Archive under accession number PRJNA890669.
