# Supplementary material for: Genetic and physical localization of a major susceptibility gene to Pyrenophora teres f. maculata in barley
Source: Theor Appl Genet. 2023 Apr 27;136(5):118. doi: 10.1007/s00122-023-04367-1 (PMC10140075; doi:10.1007/s00122-023-04367-1)
Supplement: Supplementary file 1 — Supplementary file1 (PPTX 43 KB) [file 122_2023_4367_MOESM1_ESM.pptx]

## Slide 1
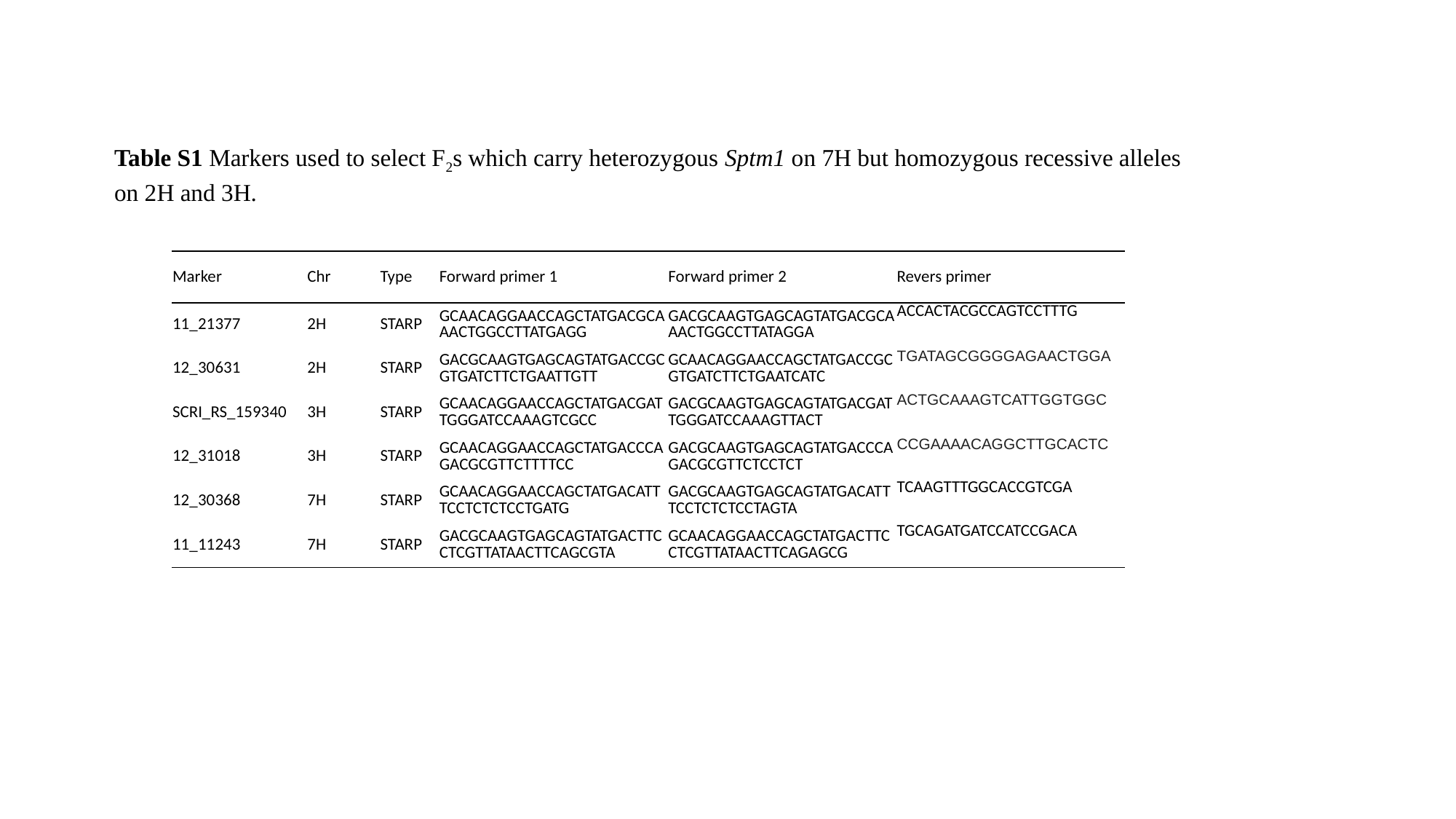

Table S1 Markers used to select F2s which carry heterozygous Sptm1 on 7H but homozygous recessive alleles on 2H and 3H.
| Marker | Chr | Type | Forward primer 1 | Forward primer 2 | Revers primer |
| --- | --- | --- | --- | --- | --- |
| 11\_21377 | 2H | STARP | GCAACAGGAACCAGCTATGACGCAAACTGGCCTTATGAGG | GACGCAAGTGAGCAGTATGACGCAAACTGGCCTTATAGGA | ACCACTACGCCAGTCCTTTG |
| 12\_30631 | 2H | STARP | GACGCAAGTGAGCAGTATGACCGCGTGATCTTCTGAATTGTT | GCAACAGGAACCAGCTATGACCGCGTGATCTTCTGAATCATC | TGATAGCGGGGAGAACTGGA |
| SCRI\_RS\_159340 | 3H | STARP | GCAACAGGAACCAGCTATGACGATTGGGATCCAAAGTCGCC | GACGCAAGTGAGCAGTATGACGATTGGGATCCAAAGTTACT | ACTGCAAAGTCATTGGTGGC |
| 12\_31018 | 3H | STARP | GCAACAGGAACCAGCTATGACCCAGACGCGTTCTTTTCC | GACGCAAGTGAGCAGTATGACCCAGACGCGTTCTCCTCT | CCGAAAACAGGCTTGCACTC |
| 12\_30368 | 7H | STARP | GCAACAGGAACCAGCTATGACATTTCCTCTCTCCTGATG | GACGCAAGTGAGCAGTATGACATTTCCTCTCTCCTAGTA | TCAAGTTTGGCACCGTCGA |
| 11\_11243 | 7H | STARP | GACGCAAGTGAGCAGTATGACTTCCTCGTTATAACTTCAGCGTA | GCAACAGGAACCAGCTATGACTTCCTCGTTATAACTTCAGAGCG | TGCAGATGATCCATCCGACA |

## Slide 2
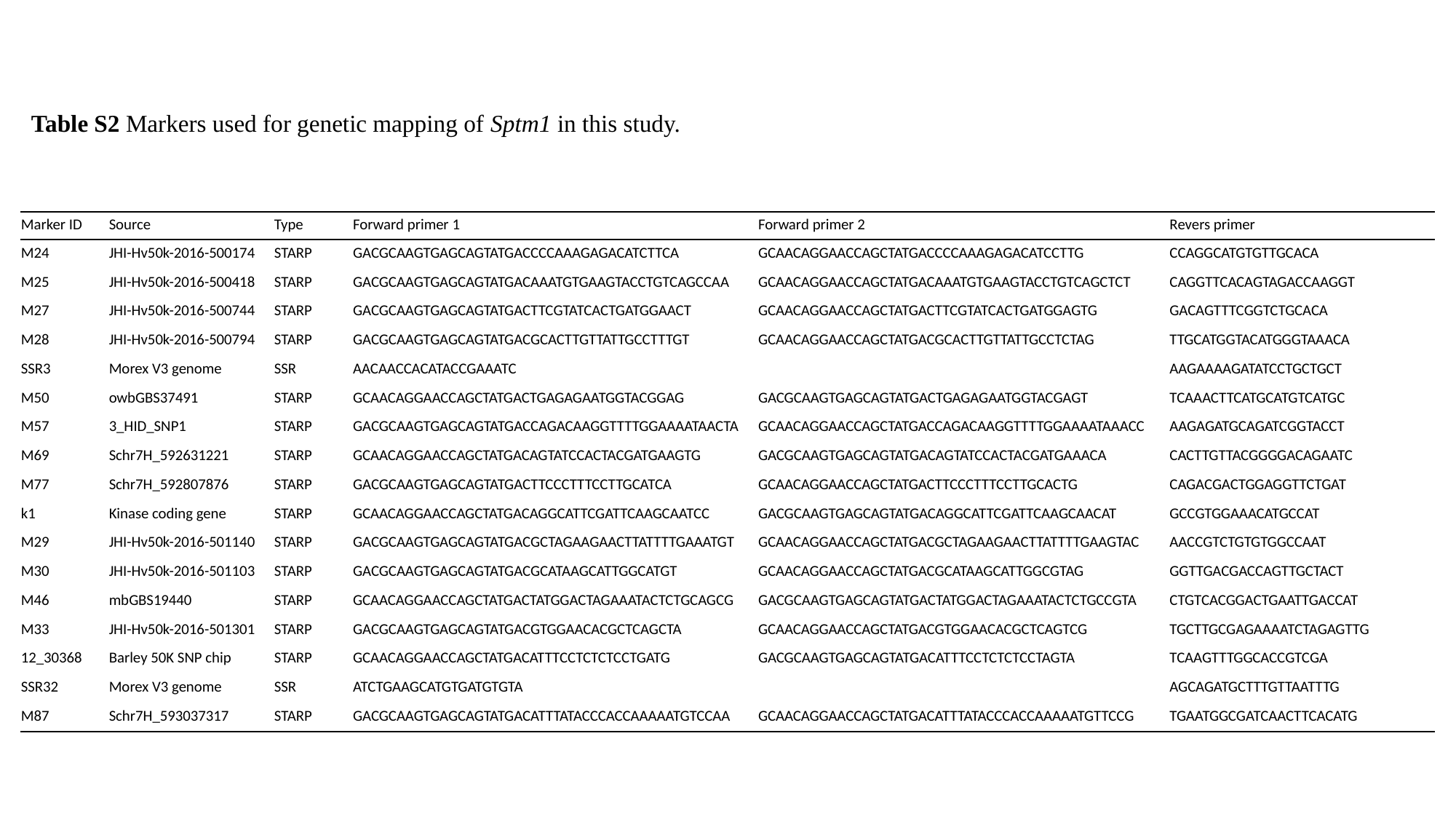

Table S2 Markers used for genetic mapping of Sptm1 in this study.
| Marker ID | Source | Type | Forward primer 1 | Forward primer 2 | Revers primer |
| --- | --- | --- | --- | --- | --- |
| M24 | JHI-Hv50k-2016-500174 | STARP | GACGCAAGTGAGCAGTATGACCCCAAAGAGACATCTTCA | GCAACAGGAACCAGCTATGACCCCAAAGAGACATCCTTG | CCAGGCATGTGTTGCACA |
| M25 | JHI-Hv50k-2016-500418 | STARP | GACGCAAGTGAGCAGTATGACAAATGTGAAGTACCTGTCAGCCAA | GCAACAGGAACCAGCTATGACAAATGTGAAGTACCTGTCAGCTCT | CAGGTTCACAGTAGACCAAGGT |
| M27 | JHI-Hv50k-2016-500744 | STARP | GACGCAAGTGAGCAGTATGACTTCGTATCACTGATGGAACT | GCAACAGGAACCAGCTATGACTTCGTATCACTGATGGAGTG | GACAGTTTCGGTCTGCACA |
| M28 | JHI-Hv50k-2016-500794 | STARP | GACGCAAGTGAGCAGTATGACGCACTTGTTATTGCCTTTGT | GCAACAGGAACCAGCTATGACGCACTTGTTATTGCCTCTAG | TTGCATGGTACATGGGTAAACA |
| SSR3 | Morex V3 genome | SSR | AACAACCACATACCGAAATC | | AAGAAAAGATATCCTGCTGCT |
| M50 | owbGBS37491 | STARP | GCAACAGGAACCAGCTATGACTGAGAGAATGGTACGGAG | GACGCAAGTGAGCAGTATGACTGAGAGAATGGTACGAGT | TCAAACTTCATGCATGTCATGC |
| M57 | 3\_HID\_SNP1 | STARP | GACGCAAGTGAGCAGTATGACCAGACAAGGTTTTGGAAAATAACTA | GCAACAGGAACCAGCTATGACCAGACAAGGTTTTGGAAAATAAACC | AAGAGATGCAGATCGGTACCT |
| M69 | Schr7H\_592631221 | STARP | GCAACAGGAACCAGCTATGACAGTATCCACTACGATGAAGTG | GACGCAAGTGAGCAGTATGACAGTATCCACTACGATGAAACA | CACTTGTTACGGGGACAGAATC |
| M77 | Schr7H\_592807876 | STARP | GACGCAAGTGAGCAGTATGACTTCCCTTTCCTTGCATCA | GCAACAGGAACCAGCTATGACTTCCCTTTCCTTGCACTG | CAGACGACTGGAGGTTCTGAT |
| k1 | Kinase coding gene | STARP | GCAACAGGAACCAGCTATGACAGGCATTCGATTCAAGCAATCC | GACGCAAGTGAGCAGTATGACAGGCATTCGATTCAAGCAACAT | GCCGTGGAAACATGCCAT |
| M29 | JHI-Hv50k-2016-501140 | STARP | GACGCAAGTGAGCAGTATGACGCTAGAAGAACTTATTTTGAAATGT | GCAACAGGAACCAGCTATGACGCTAGAAGAACTTATTTTGAAGTAC | AACCGTCTGTGTGGCCAAT |
| M30 | JHI-Hv50k-2016-501103 | STARP | GACGCAAGTGAGCAGTATGACGCATAAGCATTGGCATGT | GCAACAGGAACCAGCTATGACGCATAAGCATTGGCGTAG | GGTTGACGACCAGTTGCTACT |
| M46 | mbGBS19440 | STARP | GCAACAGGAACCAGCTATGACTATGGACTAGAAATACTCTGCAGCG | GACGCAAGTGAGCAGTATGACTATGGACTAGAAATACTCTGCCGTA | CTGTCACGGACTGAATTGACCAT |
| M33 | JHI-Hv50k-2016-501301 | STARP | GACGCAAGTGAGCAGTATGACGTGGAACACGCTCAGCTA | GCAACAGGAACCAGCTATGACGTGGAACACGCTCAGTCG | TGCTTGCGAGAAAATCTAGAGTTG |
| 12\_30368 | Barley 50K SNP chip | STARP | GCAACAGGAACCAGCTATGACATTTCCTCTCTCCTGATG | GACGCAAGTGAGCAGTATGACATTTCCTCTCTCCTAGTA | TCAAGTTTGGCACCGTCGA |
| SSR32 | Morex V3 genome | SSR | ATCTGAAGCATGTGATGTGTA | | AGCAGATGCTTTGTTAATTTG |
| M87 | Schr7H\_593037317 | STARP | GACGCAAGTGAGCAGTATGACATTTATACCCACCAAAAATGTCCAA | GCAACAGGAACCAGCTATGACATTTATACCCACCAAAAATGTTCCG | TGAATGGCGATCAACTTCACATG |

## Slide 3
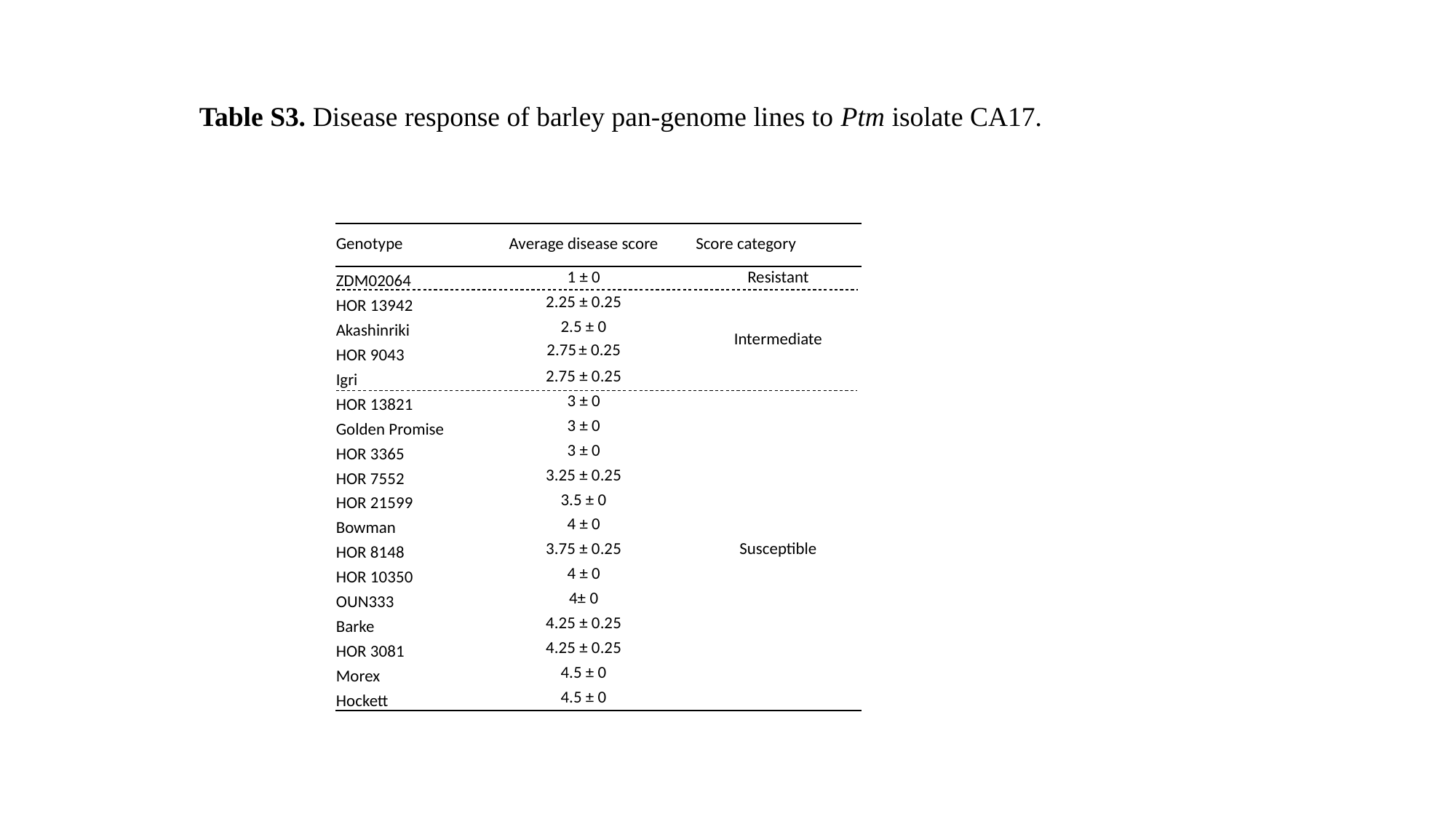

Table S3. Disease response of barley pan-genome lines to Ptm isolate CA17.
| Genotype | Average disease score | Score category |
| --- | --- | --- |
| ZDM02064 | 1 ± 0 | Resistant |
| HOR 13942 | 2.25 ± 0.25 | Intermediate |
| Akashinriki | 2.5 ± 0 | |
| HOR 9043 | 2.75 ± 0.25 | |
| Igri | 2.75 ± 0.25 | |
| HOR 13821 | 3 ± 0 | Susceptible |
| Golden Promise | 3 ± 0 | |
| HOR 3365 | 3 ± 0 | |
| HOR 7552 | 3.25 ± 0.25 | |
| HOR 21599 | 3.5 ± 0 | |
| Bowman | 4 ± 0 | |
| HOR 8148 | 3.75 ± 0.25 | |
| HOR 10350 | 4 ± 0 | |
| OUN333 | 4± 0 | |
| Barke | 4.25 ± 0.25 | |
| HOR 3081 | 4.25 ± 0.25 | |
| Morex | 4.5 ± 0 | |
| Hockett | 4.5 ± 0 | |
